# Autosomal-dominant hypotrichosis with woolly hair: Novel gene locus on chromosome 4q35.1-q35.2

**DOI:** 10.1371/journal.pone.0225943

**Published:** 2019-12-02

**Authors:** Annika E. Schlaweck, Rachid Tazi-Ahnini, F. Buket Ü. Basmanav, Javed Mohungoo, Sandra M. Pasternack-Ziach, Manuel Mattheisen, Ana-Maria Oprisoreanu, Aytaj Humbatova, Sabrina Wolf, Andrew Messenger, Regina C. Betz

**Affiliations:** 1 Institute of Human Genetics, University of Bonn, School of Medicine & University Hospital Bonn, Bonn, Germany; 2 Department of Infection, Immunity, and Cardiovascular Disease, The Medical School, University of Sheffield, Sheffield, United Kingdom; 3 Department of Dermatology, Royal Hallamshire Hospital, Sheffield, United Kingdom; 4 Department of Psychiatry, Psychosomatics, and Psychotherapy, University Hospital Würzburg, Würzburg, Germany; 5 Department of Neuropathology and Department of Epileptology, University Hospital Bonn, Bonn, Germany; German Cancer Research Center (DKFZ), GERMANY

## Abstract

Hypotrichosis simplex (HS) with and without woolly hair (WH) comprises a group of rare, monogenic disorders of hair loss. Patients present with a diffuse loss of scalp and/or body hair, which usually begins in early childhood and progresses into adulthood. Some of the patients also show hair that is tightly curled. Approximately 10 genes for autosomal recessive and autosomal dominant forms of HS have been identified in the last decade, among them five genes for the dominant form. We collected blood and buccal samples from 17 individuals of a large British family with HS and WH. After having sequenced all known dominant genes for HS in this family without the identification of any disease causing mutation, we performed a genome-wide scan, using the HumanLinkage-24 BeadChip, followed by a classical linkage analysis; and whole exome-sequencing (WES). Evidence for linkage was found for a region on chromosome 4q35.1-q35.2 with a maximum LOD score of 3.61. WES led to the identification of a mutation in the gene *SORBS2*, encoding sorbin and SH3 domain containing 2. Unfortunately, we could not find an additional mutation in any other patient/family with HS; and in cell culture, we could not observe any difference between cloned wildtype and mutant SORBS2 using western blotting and immunofluorescence analyses. Therefore, at present, *SORBS2* cannot be considered a definite disease gene for this phenotype. However, the locus on chromosome 4q is a robust and novel finding for hypotrichosis with woolly hair. Further fine mapping and sequencing efforts are therefore warranted in order to confirm *SORBS2* as a plausible HS disease gene.

## Introduction

Over the past two decades, knowledge concerning the mechanisms that control hair growth and differentiation has been increased through the discovery of a small number of disease genes, among others via next generation sequencing technologies [1–4]. Isolated forms of hair loss include e.g. monilethrix, alopecia universalis congenitalis and hypotrichosis simplex (HS, [MIM 146520, MIM 278150, MIM 146550, MIM 613981, and MIM 605389]).

HS is inherited in an autosomal-dominant or recessive manner [2], and [3] is characterized by a diffuse loss of hair, which usually begins in early childhood and progresses into adulthood. Both within and between families, the extent of scalp and body hair involvement varies, ranging from partial alopecia to a complete loss of scalp and body hair. Interestingly, some HS patients present with hair that is tightly curled and low in density. This is termed woolly hair (WH).

Available research into isolated HS with or without WH has identified mutations in around ten genes. Mutations in five of these genes—*CDSN* (MIM 602593), *APCDD1* (MIM 607479), *SNRPE* (MIM 128260), *KRT71* (MIM 608245), and *KRT74* (MIM 608248)—are responsible for autosomal dominant forms. However, mutations in these genes have been identified as the pathogenic cause in less than 20 cases/families thus accounting for only a small proportion of all HS cases. Thus the etiology of many HS cases remains unexplained.

## Material and methods

### Patient collection and DNA extraction

#### Patient collection

The study was approved by the South Sheffield Research Ethics Committee. All participants provided written informed consent. The study was conducted in accordance with the principles of the Declaration of Helsinki.

**Fig 1:**
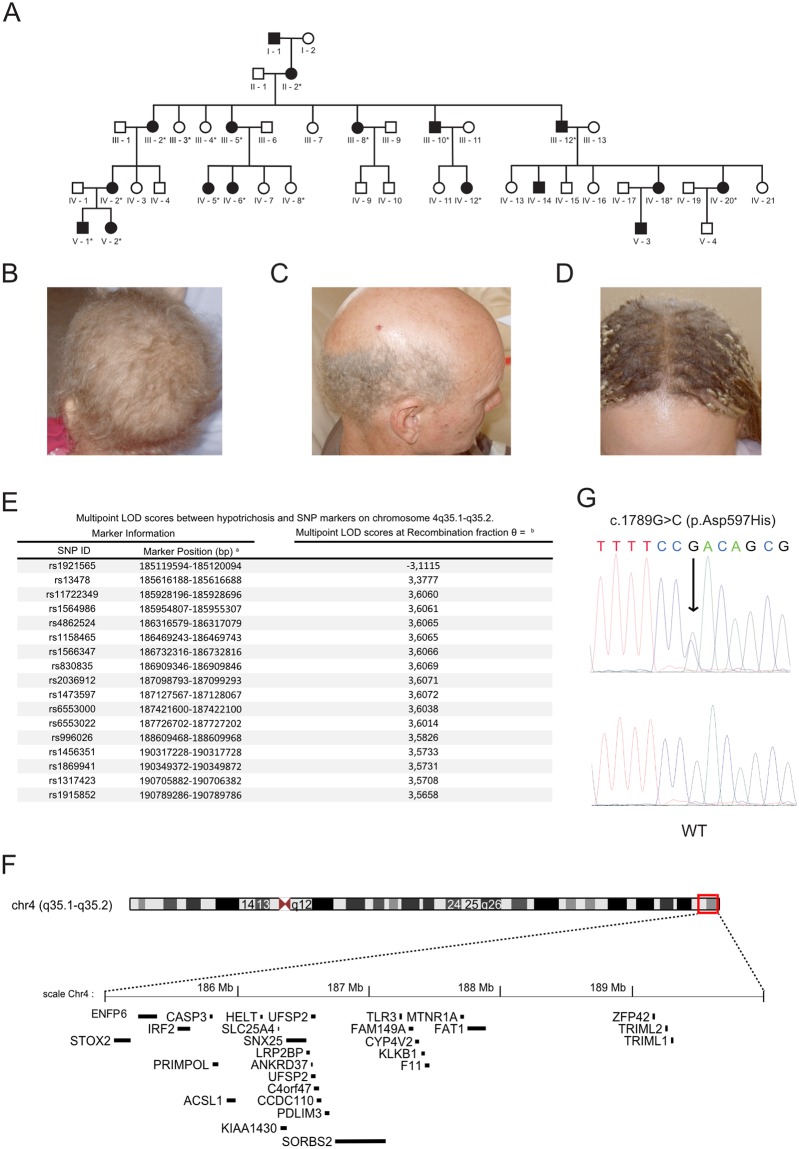
Clinical presentation, linkage analysis, candidate region, and *SORBS2* mutation. (A) Pedigree of the family. Affected family members are shown in black; circles and squares denote females and males, respectively. * indicates that DNA samples were available. (B-D) Three patients with WH accompanied HS are shown: IV-12 (B); III-12 (C); and IV-6 (D). Individual III-12 (C) independently showed male pattern baldness. The young woman displayed in (D) had applied hair extensions in order to conceal the hypotrichosis. Phenotype severity varied between family members. Mildly affected individuals showed curling of the hair and a modest reduction in hair density, which rendered the scalp visible. In most patients, these signs appeared to remain stable with increasing age although one affected individual reported a spontaneous improvement. E) Results of the multi-point linkage analysis using allegro 2.0f software. Evidence for linkage was observed on chromosome 4. The respective region spanned nearly 6 Mb between the SNPs rs1921565 and rs1915852 [chr4:184,835,760–190,789,536], with a maximum LOD score of 3.61. Notably, rs1915852 is localized at the telomeric site of the chromosome. Therefore it cannot be excluded that a candidate gene is localized at a position beyond this marker and towards the telomere. F) The region of interest on chromosome 4q35.1-q35.2 contains 28 known protein-coding genes. G) Comparison of the heterozygous c.1789G>C (p.Asp597His) mutation in *SORBS2* with the wildtype sequence. The mutation co-segregated with the hair phenotype in the pedigree.

A five generation British pedigree comprising 17 members with isolated autosomal-dominant HS with WH and 25 unaffected individuals was drawn (Fig 1A). Among the family, 17 individuals were examined in the Department of Dermatology, Royal Hallamshire Hospital, Sheffield, UK by J.M. and A.M.

#### DNA extraction

EDTA blood and buccal swap samples were collected from 17 individuals (n = 14 affected, n = 3 unaffected). DNA was extracted using standard methods.

#### Sanger sequencing

Amplicons were generated under standard polymerase chain reaction conditions (primers and conditions are available on request). Sanger sequencing was performed using the BigDye Terminator v1.1 Cycle Sequencing kit (Applied Biosystems) and an ABI 3100 genetic analyzer (Applied Biosystems). The data were analyzed using SeqMan II software (DNASTAR).

### Gene scan and classical linkage analysis

#### Gene scan

A genome-wide scan was performed including 17 individuals of the pedigree, using the HumanLinkage-24 BeadChip (Illumina, San Diego, CA). These experiments were performed on the Life & Brain Genomics platform, Bonn, Germany.

#### Linkage analysis

Data from the above mentioned gene scan were used. The graphical user interface ALOHOMORA was used for data management and quality control [5]. After quality control, a total of 5.553 markers were included in the analysis. Marker allele frequencies were calculated using founders from the “CEU” subgroup of the HapMap3 dataset (obtained through http://zzz.bwh.harvard.edu/plink/dist/hapmap_CEU_r23a_filtered.zip). Using the allegro 2.0f software, multi-point parametric LOD scores were calculated for HS with WH and each marker locus under three assumptions: (i) autosomal-dominant inheritance with complete penetrance; (ii) disease allele frequency of 10^−5^; and (iii) equal allele frequencies for each marker.

### Whole exome sequencing

The index patient was exome sequenced by Oxford Gene Technology’s Genefficiency Sequencing Service. Genomic DNA (2 μg) was fragmented and enriched for human exonic sequences using the Human All Exon V5 Agilent Sure Select kit (Agilent Technologies) using the manufacturers protocol, and sequenced on the Illumina HiSeq 2000 platform using Truseq (v3 Chemistry) (Illumina) to generate 100 base paired-end reads. Fastq files were mapped to the reference human genome (hg19/GRCh37) using the Burrows-Wheeler Aligner (BWA) package (v0.6.2) [6]. Local realignment of the mapped reads around potential insertion/deletion (indel) sites was carried with the Genome Analysis Tool Kit (GATK) v1.6 [7]. Duplicate reads were marked using Picard v1.8 and BAM files were sorted and indexed with SAM tools v0.1.18 [8]. Approximately 12 and 14 GB of sequence data was generated for these samples and a minimum of 90.83% and 80.54% of the targeted exome was covered to a depth of at least 20× and 30× coverage, respectively. We filtered the variants for high-quality heterozygous, novel variants [defined against dbSNP 132 inclusion] and are deleterious based on either of the SIFT, Polyphen and Condel predictions.

Data cannot be shared publicly. The data underlying the results presented in the study are available from the corresponding author or from Dr. Per Hoffmann (Email: phoffmann@lifeandbrain.com).

### Cloning

For this purpose, a pcDNA3.1 vector containing the sequence for *SORBS2* transcript variant 2 with a C-terminal V5 tag was used (GenScript, Piscataway, NJ). Using the QuikChange II Site-Directed Mutagenesis kit (Agilent, Santa Clara, CA; primers and conditions are available on request), the mutant construct was generated. HEK293T cells were maintained at 37°C (5% CO_2_) in DMEM (Lonza) supplemented with 10% FCS (Life Technologies), 1% Penicillin-Streptomycin (10,000 U ml^-1^, Life Technologies) and 1% Amphotericin B (250 μg ml^-1^, Life Technologies). Cells were cultured in 10 cm Petri dishes and on coverslips in 12-well plates for western blotting and immunofluorescence analysis, respectively and transiently transfected with the wild-type (WT) and mutant constructs using the Lipofectamine2000 Transfection Kit (Life Technologies) according to manufacturer’s instructions. For each 10 cm plate, the following amounts of reagents were used: 10 μg plasmid, 700 μl Opti-MEM (Life Technologies) and 15 μl Lipofectamine2000. For each well in a 12-well plate, 0.5 μg of plasmid, 50 μl Opti-MEM and 1 μl Lipofectamine2000 were used. Cells were harvested 48 h post-transfection.

### Western blotting

Cells were collected in ice-cold PBS and centrifuged at 150 x g at 4°C for 10 min. The cell pellets were re-suspended in 800 μl lysis buffer (HEPES 50mM, NaCl 80mM, Protease Inhibitor Cocktail 1%, TritonX 1% up to 10ml Millipore), incubated on ice for 30 min and sonicated 10 times for 10 sec, with 10 sec breaks on ice. After centrifugation at 10,500 x g for 10 min at 4°C, the supernatants were transferred to clean tubes. Purified lysates were mixed with NuPAGE-LDS Sample Buffer (4x, Invitrogen, NP0007) and Reducing agent (10x, Life Technologies, NP0004). The mixtures were boiled at 95°C for 5 min. After protein separation on 4–12% NuPAGE-Bis-Tris gels (Life Technologies, NP0322BOX) at 200V for 45 min, the proteins were blotted on PVDF membrane (Invitrogen) at 32V for 2 hrs. Western blotting was carried out using the WesternBreeze chemiluminescent kit (Invitrogen) according to manufacturer’s instructions. Immunoblotting was performed using mouse monoclonal anti-V5 (1:5.000, Thermo Fisher, R960-25) and anti-GAPDH (1:10.000, Sigma Aldrich, G8795) antibodies. Membranes were then exposed to an X-ray film for 5–10 min.

### Immunofluorescence analyses

Transiently transfected HEK293T cells grown on coverslips were fixed for 10 min with 4% PFA at RT, washed with 1x PBS for 3x5 min, permeabilized for 10 min with PBS containing 1% Triton X-100 and blocked for 40 min in PBS containing 3% bovine serum albumin. Cells were incubated at first with mouse anti-V5 antibody (1:200, Thermo Fisher, R960-25). After several washing steps, cells were incubated with goat anti-mouse Cy3 (1:400, Life Technologies, A10521), Alexa Fluor^®^ 647 Phalloidin (1:100, Life Technologies, A22287) and DAPI (1:100, Sigma, D9542). The nuclei were stained with DAPI. Images were captured with CFI Plan APO IR 60x water immersion objective (N/A 1.27) using a laser scanning Nikon A1/Ti confocal microscope and a Nikon NIS-Elements 4.0 acquisition software. From Z-stacks (Z-step 0.275um) maximum projection intensity were generated. ImageJ was used for the analyses by applying the same brightness and contrast thresholds to all data. Two to three coverslips were analyzed per construct at each transfection.

## Results

The present report describes a five generation British pedigree comprising 17 members with autosomal-dominant isolated HS with WH and 25 unaffected individuals (Fig 1A). The affected individuals presented in infancy with sparse WH and varying degrees of body/scalp involvement (Fig 1B–1D). Mildly affected individuals showed curling of the hair and a modest reduction in hair density. In five of the 14 affected individuals examined for the present study, keratosis pilaris of the arms was observed. Further medical history was unremarkable.

To identify the molecular cause of HS with WH in this family, blood and buccal swap samples were collected from 17 individuals (n = 14 affected, n = 3 unaffected). DNA was extracted using standard methods (described in Methods).

Investigation of the five known genes for autosomal dominant HS by targeted gene sequencing revealed no disease causing mutation. So we aimed to search for a novel gene that is causative for HS with WH in this family and applied two complementary approaches: i) a classical linkage analysis was performed using genome-wide SNP data from 17 related individuals to identify the chromosomal regions co-segregating with the phenotype and (ii) whole exome-sequencing was performed in the index patient to obtain the full spectrum of low-frequency and rare sequence variants in the protein coding regions of the genome. Evidence for linkage was found for a region on chromosome 4q35.1-q35.2. This was bounded by the SNPs rs1921565 and rs1915852. The maximum LOD score was 3.61 (Fig 1E).

The region of interest on chromosome 4q contains 28 known protein-coding genes (Fig 1F). None of these genes appeared to be of relevance for a hair-related phenotype, therefore, we had to assume all of these genes as putative candidate genes for the observed phenotype. Investigation of the novel and deleterious variants in these genes from the exome data led to the identification of the following two heterozygous missense mutations: (i) c.1789G>C;p.Asp597His in *SORBS2* (MIM 616349), encoding sorbin and SH3 domain containing 2, a cytoskeletal scaffolding protein (Fig 1G); and (ii) c.599G>A;p.Cys200Tyr in *F11* (MIM 264900), encoding coagulation factor XI. Both mutations were validated by Sanger sequencing and they co-segregated with the hair phenotype. Since mutations in *F11* cause a congenital factor XI deficiency, four affected family members were reexamined. Their FXI levels were below 43.2 (NR 60–150 U/dl). Thus Factor XI deficiency was confirmed as an additional diagnosis, which was probably independent of the hair phenotype. We, therefore, concluded that the hair phenotype may be attributable to the mutation in *SORBS2*.

**Fig 2:**
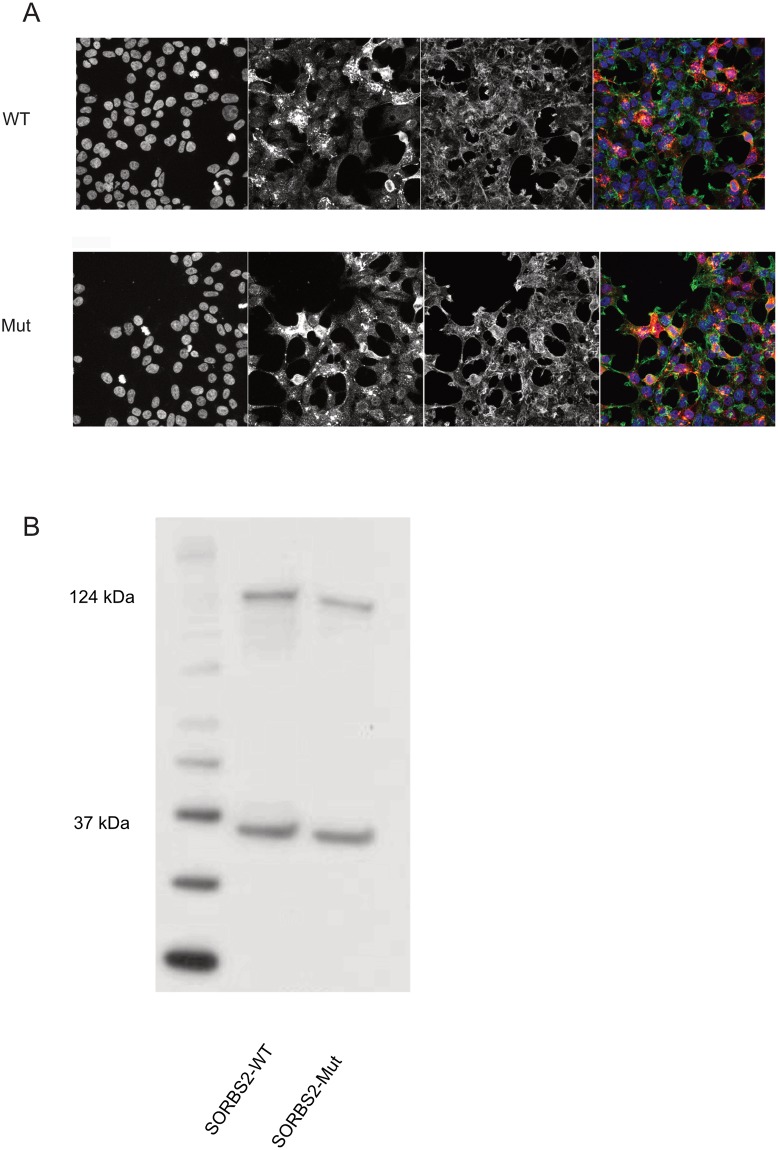
Immunoblotting and immunofluorescence analyses in HEK293T cells. (A) Immunofluorescence analysis revealed a similar actin cytoskeleton structure in HEK293T cells transiently transfected with WT or mutant SORB2-V5 constructs. In addition, no significant difference between the subcellular localization of WT and mutant SORBS2 was observed. Scale bars: 50 μm. (B) Immunoblotting of transiently transfected HEK293T lysates revealed no difference between the translation of WT and mutant SORBS2-V5. Translation of the WT and mutant SORBS2 was observed at around 124 kDA. GAPDH (37 kDA) served as the loading control.

The *SORBS2* mutation was located in exon 13, which is only a part of three among many splice forms of this gene. To support the role of *SORBS2* in HS with WH, we initially performed targeted sequencing of *SORBS2* in more than 30 additional HS/WH cases and identified no additional pathogenic mutations in these individuals. Seeking for additional support for disease causality, we investigated if the identified *SORBS2* missense mutation confers a pathogenic effect detectable at the level of protein expression or subcellular localization. Accordingly, HEK293T cells were transiently transfected with mammalian expression constructs encoding for wildtype (WT) and missense mutant of SORBS2 fused to a V5 tag. SORBS2 protein is known to interact with actin and other cytoskeletal proteins and is suggested to be in involved in actin/cytoskeletal organization [9]. Therefore, the cells were double immunostained for SORBS2 and actin filaments by an anti-V5 antibody and the high affinity F-actin probe Phalloidin conjugated to a fluorescent dye, respectively. Confocal microscopy analysis did not reveal any differences between the WT and mutant SORBS2 in terms of i) their subcellular localization or ii) the cytoskeletal structure of cells expressing either of these two proteins (Fig 2A). To check for any effect of the missense mutation on the length or expression of the protein we performed Western blotting (WB) which also failed to detect any differences between WT and mutant forms of SORBS2 (Fig 2B).

## Discussion

Genome-wide linkage analysis complemented with exome sequencing revealed heterozygous mutations in the *F11* and *SORBS2* genes segregating with the phenotype in the examined family. No other strong candidate genes in the linkage region were identified to harbor deleterious mutations.

The mutation in *F11* caused an already known subclinical coagulopathy with slightly decreased FXI levels. A hair phenotype had not been described in this context. Therefore, we speculated on the causative relation between the *SORBS2* mutation and the hereby presented hair phenotype.

SORBS2 is also known as ARGBP2, and is a cytoskeletal scaffolding protein that was initially discovered in 1997. The respective report found that ARGBP2 interacted with the c-Arg and c-Abl kinases, thereby linking them to the actin cytoskeleton [10]. Subsequent research demonstrated that SORBS2 is involved in a number of cytoskeleton associated cell signaling processes, such as migration and adhesion [11–13]. To date, no study has implicated SORBS2 in hair loss, or investigated its potential role in hair or skin tissue development. However, previous authors investigated the potential influence of SORBS2 on senescence in primary human keratinocytes in order to determine its previously reported role in carcinogenesis [14]. This study showed that while ectopic expression of SORBS2 induced senescence, endogenous expression showed a pronounced increase during near senescent passages in *in vitro* cultured primary keratinocytes [14].

At present, *SORBS2* cannot be considered a definite disease gene for HS with WH, given the lack of: (i) additional data concerning the relationship between SORBS2 and hair morphogenesis; (ii) other HS patients/pedigrees with *SORBS2* mutations; and (iii) evidence for pathogenicity of the identified mutation in the present WB and IF experiments. However, the locus on chromosome 4q is a robust and novel finding for HS with WH. Further fine mapping of the region (e.g. by whole genome sequencing efforts) in this family as well as sequencing of additional unexplained cases of HS with woolly hair are warranted to confirm *SORBS2*—or any other gene from the linkage region—as a novel disease gene for autosomal-dominant HS with WH.
